# Addition of cariogenic pathogens to complex oral microflora drives significant changes in biofilm compositions and functionalities

**DOI:** 10.1186/s40168-023-01561-7

**Published:** 2023-06-01

**Authors:** Yuan Liu, Scott G. Daniel, Hye-Eun Kim, Hyun Koo, Jonathan Korostoff, Flavia Teles, Kyle Bittinger, Geelsu Hwang

**Affiliations:** 1 Department of Preventive and Restorative Sciences, School of Dental Medicine, University of Pennsylvania, Philadelphia, PA 19104 USA; 2 Department of Gastroenterology, Hepatology, and Nutrition, Children’s Hospital of Philadelphia, Philadelphia, PA 19104 USA; 3 Department of Orthodontics, School of Dental Medicine, University of Pennsylvania, Philadelphia, PA 19104 USA; 4 Center for Innovation & Precision Dentistry, School of Dental Medicine, School of Engineering and Applied Sciences, University of Pennsylvania, Philadelphia, PA 19104 USA; 5 Department of Periodontics, School of Dental Medicine, University of Pennsylvania, Philadelphia, PA 19104 USA; 6 Department of Basic & Translational Sciences, School of Dental Medicine, University of Pennsylvania, Philadelphia, PA 19104 USA; 7 Department of Chemical and Biomolecular Engineering, College of Engineering, Yonsei University, Seoul, 03722 Republic of Korea

**Keywords:** Oral biofilms, Human saliva, *Streptococcus mutans*, *Candida albicans*, Dietary sugars, Composition, Functionality

## Abstract

**Background:**

Dental caries is a microbe and sugar-mediated biofilm-dependent oral disease. Of particular significance, a virulent type of dental caries, known as severe early childhood caries (S-ECC), is characterized by the synergistic polymicrobial interaction between the cariogenic bacterium, *Streptococcus mutans*, and an opportunistic fungal pathogen, *Candida albicans*. Although cross-sectional studies reveal their important roles in caries development, these exhibit limitations in determining the significance of these microbial interactions in the pathogenesis of the disease. Thus, it remains unclear the mechanism(s) through which the cross-kingdom interaction modulates the composition of the plaque microbiome. Here, we employed a novel ex vivo saliva-derived microcosm biofilm model to assess how exogenous pathogens could impact the structural and functional characteristics of the indigenous native oral microbiota.

**Results:**

Through shotgun whole metagenome sequencing, we observed that saliva-derived biofilm has decreased richness and diversity but increased sugar-related metabolism relative to the planktonic phase. Addition of *S. mutans* and/or *C. albicans* to the native microbiome drove significant changes in its bacterial composition. In addition, the effect of the exogenous pathogens on microbiome diversity and taxonomic abundances varied depending on the sugar type. While the addition of *S. mutans* induced a broader effect on Kyoto Encyclopedia of Genes and Genomes (KEGG) ortholog abundances with glucose/fructose, *S. mutans*-*C. albicans* combination under sucrose conditions triggered unique and specific changes in microbiota composition/diversity as well as specific effects on KEGG pathways. Finally, we observed the presence of human epithelial cells within the biofilms via confocal microscopy imaging.

**Conclusions:**

Our data revealed that the presence of *S. mutans* and *C. albicans*, alone or in combination, as well as the addition of different sugars, induced unique alterations in both the composition and functional attributes of the biofilms. In particular, the combination of *S. mutans* and *C. albicans* seemed to drive the development (and perhaps the severity) of a dysbiotic/cariogenic oral microbiome. Our work provides a unique and pragmatic biofilm model for investigating the functional microbiome in health and disease as well as developing strategies to modulate the microbiome.

Video Abstract

**Supplementary Information:**

The online version contains supplementary material available at 10.1186/s40168-023-01561-7.

## Background

Dental caries (tooth decay) remains one of the most prevalent and costly biofilm-dependent oral diseases that affect both children and adults [1]. Of particular significance is a virulent type of tooth decay known as severe early childhood caries (S-ECC), which frequently afflicts underprivileged preschool children, causing enormous health and economic burdens [2]. Left untreated, S-ECC can lead to the rampant destruction of mineralized tooth structure, marked pain, and systemic complications. Ultimately, this can have a significant impact on the nutritional and linguistic development of affected children [3]. Therefore, further understanding of the etiology of S-ECC is urgently needed in order to develop novel approaches to prevent or intervene in the progression of the disease.

A unique clinical feature of S-ECC is the synergistic polymicrobial interaction between the cariogenic bacterium, *Streptococcus mutans* (Sm), and an opportunistic fungal pathogen, *Candida albicans* (Ca). Evidence from prior in vitro and in vivo studies revealed that Ca interacts with Sm forming inter-kingdom biofilms in the presence of sucrose, reinforcing biofilm pathogenesis, and enhancing the progression of S-ECC [4–11]. Such dietary sugar-driven biofilm accumulation on tooth surfaces and localized acidification cause deleterious alteration of the microbial community. Furthermore, this process disrupts enamel mineral homeostasis, thereby amplifying the severity of tooth decay [12–15].

Based on advances in next-generation 16S rRNA sequencing technology, a growing number of studies have demonstrated that the bacterial composition of plaque derived from subjects with caries is distinct compared to that of caries-free subjects [16–20]. One recent case–control cross-sectional study revealed that the presence of oral Ca is associated with a highly acidogenic and acid-tolerant bacterial community in S-ECC, with an increased abundance of *Streptococcus* species, especially Sm [21]. However, it is critical to note that these cross-sectional studies have limitations regarding the determination of the significance of microbial interactions between specific organisms in the pathogenesis of the disease [22]. As such, while the important roles of Ca and Sm in caries development have been largely acknowledged, the mechanism(s) through which the cross-kingdom interaction modulates the composition of the plaque microbiome and caries severity remains unclear.

Given the complexity of the human caries-associated plaque microbiome and the ethical dilemmas accompanying in vivo studies, various in vitro models have been proposed to study oral biofilm formation. For example, natural resources such as human saliva and dental plaque have been used as the inoculum in a range of experimental models, known as microcosm biofilm models, to evaluate the composition and prevalence of microbial species related to dental caries within a complex plaque-like community under different growth conditions [23–26]. However, previous studies were mainly focused on the effects of different growth conditions or dietary sugars on regulating oral microecology rather than investigating how exogenous pathogens could impact oral microbiota.

In recognition of these issues, we developed a novel ex vivo saliva-derived microcosm biofilm system that employs human whole saliva not only as an inoculum but also as a growth medium instead of artificial saliva or nutrient broth. To determine the mechanism through which key pathogens drive the ecological and microbial processes that underlie the transition from a healthy to a disease-associated oral microbiome, salivary inocula containing Ca or Sm, or both organisms in combination or none, were used. The influence of dietary sugars (i.e., sucrose and glucose/fructose) was also evaluated to assess the impact on the composition and behavior of the ex vivo biofilms. In this study, we utilized shotgun whole metagenome sequencing to identify the members of the ex vivo microbial community and their functionalities under various conditions. We observed that the presence of Ca and Sm, alone or in combination, as well as the addition of different sugars, induced unique alterations in both the composition and functional attributes of the biofilms. This new unique and pragmatic ex vivo microcosm biofilm model combined with an advanced sequencing method may deepen our understanding of the etiology and pathogenesis of S-ECC.

## Methods

### Saliva collection

This present study was reviewed and approved by the Institutional Review Board of the University of Pennsylvania (protocol #818549). Written informed consent was obtained from healthy volunteers in the study. Saliva was collected by chewing unflavored paraffin wax for two purposes: inoculum and coating the hydroxyapatite (HA) discs. For HA disc coating, collected saliva was centrifuged (5500 × *g*, 4 °C, 10 min) and filtered using 0.22 µm polyethersulfone, ultra-low binding protein filters (Millipore, Billerica, MA) [10, 11]. Filtered saliva was then kept in a 4 °C refrigerator until use to minimize the precipitation of salivary proteins [27].

### Microorganisms and culture conditions

*Streptococcus mutans* UA159 (ATCC 700610), a virulent cariogenic pathogen, and *Candida albicans* SC5314, a well-characterized fungal strain, were used for the ex vivo biofilm model. Both strains were grown to mid-exponential phase (optical densities at 600 nm of 1.0 and 0.8 for *S. mutans* and *C. albicans*, respectively) in ultra-filtered (10-kDa molecular-mass cutoff; Millipore, Billerica, MA, USA) tryptone-yeast extract (UFTYE) broth with 1% (wt/vol) glucose at 37 °C and 5% CO_2_ [10].

### Ex vivo biofilm model

Biofilms were formed on saliva-coated hydroxyapatite (sHA) discs (surface area = 2.7 ± 0.2 cm^2^, Clarkson Chromatography Products, Inc., South Williamsport, PA) vertically suspended in 24-well plates using a custom-made disc holder, mimicking the smooth surfaces of the pellicle-coated tooth. Each HA disc was coated with the filtered saliva for 1 h at 37 °C. Each sHA was then inoculated with (i) whole saliva, (ii) whole saliva with approximately 2 × 10^6^ colony forming units (CFU) of *S. mutans*/mL, (iii) whole saliva with approximately 2 × 10^4^ CFU of *C. albicans*/mL, and iv) whole saliva with both ~ 2 × 10^6^ CFU of *S. mutans*/mL and ~ 2 × 10^4^ CFU of *C. albicans*/mL [10, 11]. The concentrations for *S. mutans* and *C. albicans* used in this study are based on clinical findings from children with S-ECC whereby the detection levels of *S. mutans* and *C. albicans* in plaque samples from S-ECC children are approximately 10^6^ and 10^4^ CFU/mL, respectively [28–30]. The whole saliva was supplemented with 1% (wt/vol) sucrose or 0.5% (wt/vol) glucose and 0.5% (wt/vol) fructose. The discs were incubated at 37 °C with 5% CO_2_ without disturbance for the first 18 h. Subsequently, the cultured saliva medium was replaced with fresh whole saliva supplemented with either sugar type twice at 18 h and 28 h. At 42 h, the biofilms and planktonic phases were collected for DNA extraction and shotgun metagenomic sequencing. In a separate experiment, we examined the spatial organization of biofilms using confocal laser scanning microscopy (CLSM). Briefly, bacterial cells were stained with 2.5 µM SYTO 9 green-fluorescent nucleic acid stain (485/498 nm; Molecular Probes) and *C. albicans* cells were stained with concanavalin A (ConA) lectin conjugated with tetramethylrhodamine at 40 µg/mL (555/580 nm; Molecular Probes), while exopolysaccharides were labeled with 1 µM Alexa Fluro 647-dextran conjugate (647/668 nm; Molecular Probes) as detailed previously [10, 11] DAPI (340/460 nm; Molecular Probes) at 1 µg/mL was used to stain human cells. Confocal images were obtained using an upright single-photon confocal microscope (LSM800, Zeiss) with a × 20 (numerical aperture, 1.0) water objective. All experiments were conducted four times; each condition contains 4 biological samples.

### DNA sequencing

DNA extraction was performed using the Qiagen DNeasy PowerSoil kit. After extraction, 1 ng of DNA was used to generate shotgun metagenomics with the NexteraXT kit. Libraries were sequenced on an Illumina HiSeq 2500 in High Output mode, to produce paired-end 125 bp sequence reads. Negative control samples, including DNA extraction blanks and DNA-free water, were included alongside experimental samples. Positive control samples, consisting of a laboratory-generated mock DNA community (*Vibrio campbellii*, *Cryptococcus diffluens*, and lambda phage) were also prepared and sequenced with the experimental samples.

### Statistical analysis

Shotgun metagenomic sequence data were processed using the Sunbeam bioinformatics pipeline [31]. Bacterial and archaeal taxon abundances were estimated using Kraken [32]. The abundance of gene orthologs was assessed by alignment to the KEGG database [33]. Pathway abundances were calculated using a weighted sum of the orthologs assigned to each pathway. When investigating orthologs in changed pathways, we filtered for orthologs that had at least 1000 counts across samples. Statistical analysis was performed in R (Version 4.0.3) [34]. The PERMANOVA test was used to detect differences based on the Bray–Curtis distance between samples [35]. Diversity was computed using the *vegan* package version 2.5–7 [36]. Following the logit transformation of relative abundances, linear mixed-effects models were used to detect differences in gene and taxon abundance between sample groups, and repeated measurements were accounted for as a random effect. *P* values from multiple testing procedures were corrected to control for a false discovery rate of 5%.

## Results

**Fig. 1:**
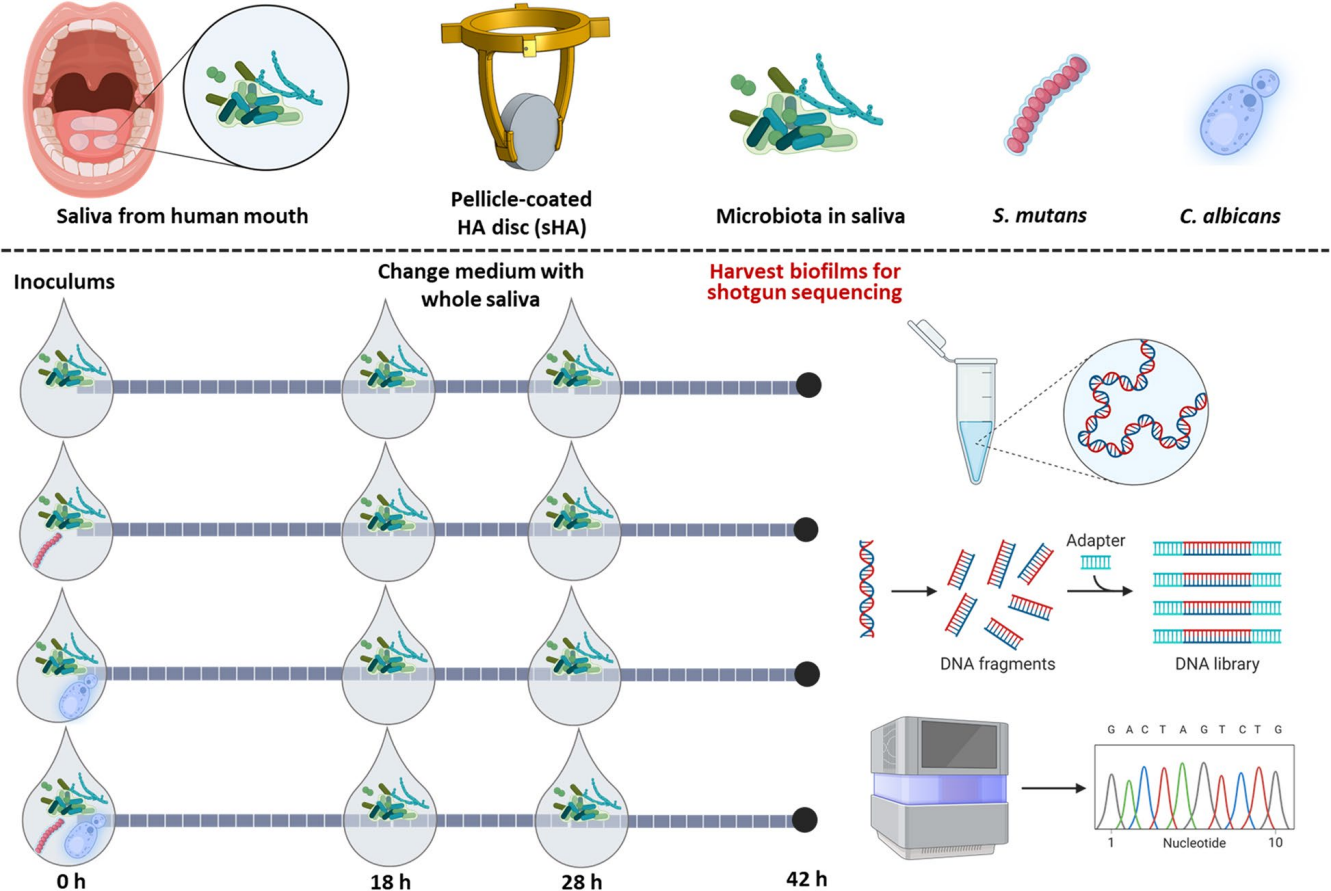
Scheme showing a novel ex vivo microcosm model using saliva-coated hydroxyapatite (sHA) disc. Saliva from a healthy donor was inoculated with or without the addition of *S. mutans* and/or *C. albicans*, and incubated on an sHA disc for 42 h. Then, the samples from the planktonic phase and biofilm under each condition were subjected to shotgun whole metagenome sequencing to identify both the composition and functional attributes of the biofilms. Created with BioRender.com

Our overarching hypothesis is that the exogenous introduction of Ca and/or Sm to a human oral microflora devoid of these pathogens will, under sugar-rich diet conditions, trigger a set of biological and functional changes within the biofilm as well as a shift in its microbial composition. To test this, it was first necessary to screen saliva from multiple donors to identify samples lacking Ca or Sm. We used ChromAgar and Mitis Salivarius Agar plus Bacitracin (MSB) to identify Ca and Sm*,* respectively [7]. Based on the initial screening, we found that most of the saliva samples contained a range of Sm populations (10^2^ to 10^4^ CFU/mL) but not Ca. Notably, one saliva donor lacked both Ca and Sm (Table S1). We then performed ex vivo biofilm experiments by using non-clarified whole saliva from the donor not carrying both Ca and Sm with (i) no external organisms added, addition of (ii) Ca (10^4^ CFU/mL) alone, (iii) Sm (10^6^ CFU/mL) alone, or (iv) both Ca (10^4^ CFU/mL) and Sm (10^6^ CFU/mL) (Fig. 1). The experiment was conducted using a sucrose-dependent (1% (wt/vol) sucrose) or a sucrose-independent model (0.5% (wt/vol) each of glucose and fructose; equimolar monosaccharide carbon-source). Sucrose is a unique cariogenic disaccharide because it is both fermentable and a substrate for extracellular polymeric substances (EPS) glucan synthesis [37, 38]; glucose and fructose are also fermentable but unable to generate EPS.

### Biofilm microbiota and functional pathways are distinct from the planktonic phase

We collected all inocula as well as 4 planktonic and biofilm samples from each combination of inoculum, carbon source, and sample type at the endpoint of the experiments (42 h, *n* = 64). All samples were shotgun sequenced, yielding a total of 509 million reads. After filtering out low quality, low complexity, and human reads, 439 million reads were used for further analysis. Nucleotide base quality had an average Phred score of 36.

**Fig. 2:**
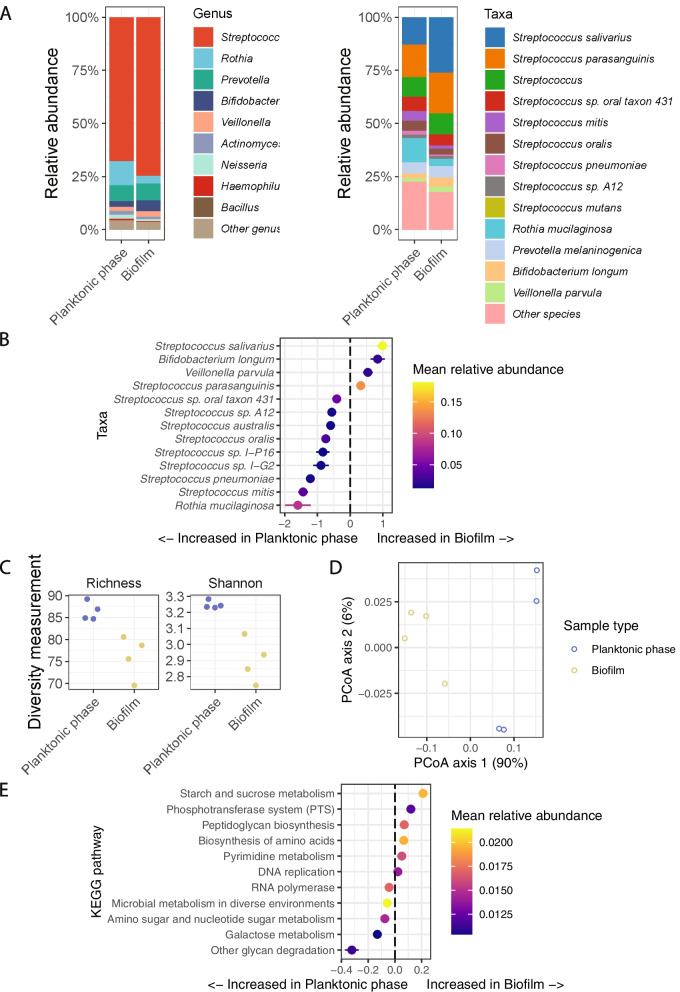
Composition of most abundant taxa in planktonic phase and biofilm in saliva with glucose/fructose as carbon source. **A** Bacteria relative abundance within the planktonic phase and biofilm at genus and species level. **B** Difference in log2-transformed abundances of bacterial species in biofilm compared to the planktonic phase. Bars indicate ± standard error in the linear mixed-effects model. Taxa shown are those that had FDR < 0.05 in the linear mixed-effects model. **C** Alpha-diversity differences between biofilm and planktonic phase. **D** Principle coordinates analysis plot of Bray–Curtis dissimilarity between samples. *X* and *Y* axis show the percentage of total variance captured. **E** Difference in log2-transformed abundances of KEGG pathways in biofilm compared to the planktonic phase. Bars indicate ± standard error in the linear mixed-effects model. Pathways shown are those that had FDR < 0.05 in the linear mixed-effects model

Initially, we restricted our analysis to samples that had glucose/fructose as a carbon source and with no Ca or Sm added. At the genus level, *Streptococcus*, which is known as the predominant microbe within the total microflora of a healthy oral cavity [39], was also the most abundant organism in both planktonic and biofilm samples, averaging 68% and 74% relative abundance, respectively (Fig. 2A). The most abundant species within the *Streptococcus* genus were *S. salivarius* and *S. parasanguinis*. We compared the relative abundances of the top species between the planktonic phase and biofilm to identify differences (Fig. 2B). Four species were increased in the biofilm, particularly *S. salivarius*, *Bifidobacterium longum*, and *Veillonella parvula* (false discovery rate (FDR) = 1.6e − 03, 0.011, and 0.012, respectively) while nine species were decreased.

The analysis of diversity showed the bacterial community structures were distinct between sample types. In terms of alpha-diversity, the raw number of species (richness) and the Shannon index, a measurement of species evenness, decreased in the biofilms compared to the planktonic phases (*P* = 7.8E − 3 and 2.4E − 3, respectively; Fig. 2C). Analysis of the beta-diversity, using Bray–Curtis distances, indicated the bacterial community composition was different between biofilms and planktonic phases (*P* = 0.022, PERMANOVA test; Fig. 2D).

Seeing the changes in community composition, we sought to understand the differences in bacterial gene function in the biofilm versus the planktonic phase. We found that 11 KEGG pathways were different depending on the sample type (Fig. 2E). Notably, sugar uptake via the phosphotransferase system was considerably amplified (FDR = 0.008), which might lead to a significant surge in the subsequent catabolic process (starch and sucrose metabolism) within the biofilm (FDR = 0.004). Conversely, other glycan degradation experienced the highest increase in the planktonic phase (FDR = 0.005). In addition, certain anabolic processes such as peptidoglycan biosynthesis and biosynthesis of amino acids were found to be heightened within the biofilms (FDR = 0.017 and 0.005, respectively). In contrast, two other sugar-related pathways, amino/nucleotide sugars, and galactose metabolism were in greater abundance in planktonic phases (FDR < 0.001 and FDR = 0.007, respectively). In summary, increased abundance of some *Streptococcal* species, decreased diversity, and increased starch and sucrose metabolism in the biofilm were observed when saliva was used as inocula and glucose/fructose was supplemented as a carbon source.

### Addition of pathogens and the supplemented carbon sources drive significant changes in microbiome diversity, taxonomic abundances, and biofilm compositions

**Fig. 3:**
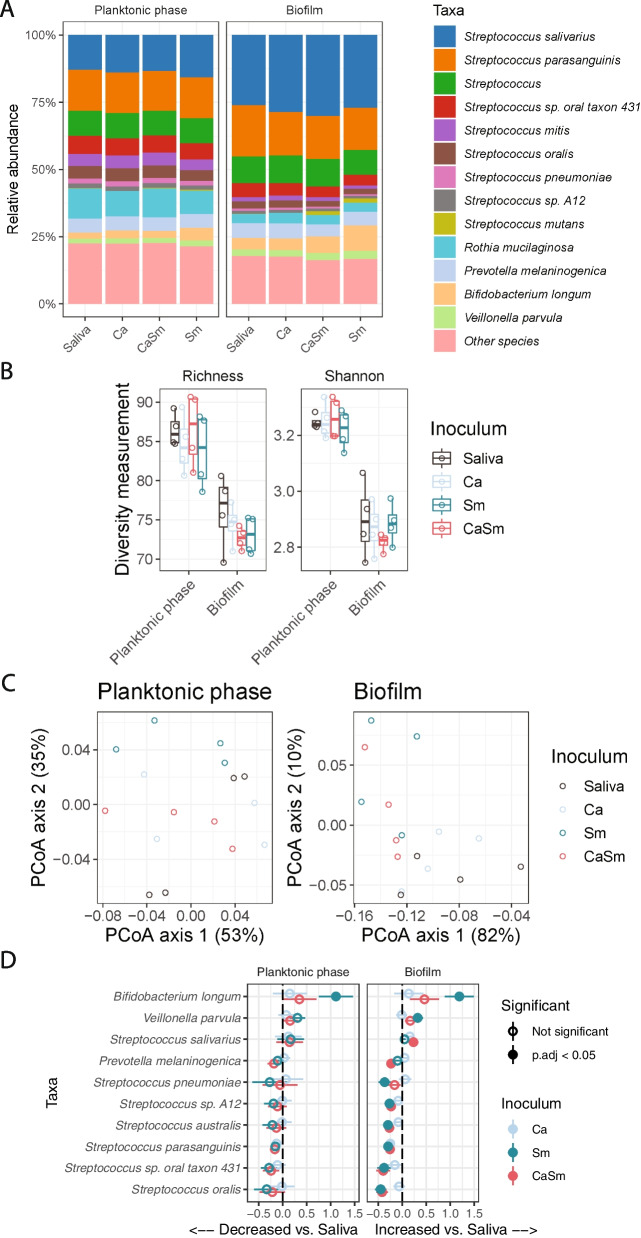
Effects of introducing opportunistic pathogens on planktonic phase and biofilm microbiota with glucose/fructose. **A** Bacteria relative abundance within planktonic phases and biofilms when introducing *S. mutants* and/or *C. albicans*. **B** Alpha diversity of planktonic and biofilm samples with different pathogens. **C** Principle coordinates analysis plot of Bray–Curtis dissimilarity between samples with *S. mutants* and/or *C. albicans*. *X* and *Y* axis show the percentage of total variance captured. D) Difference in log2-transformed abundances of bacterial species as a result of adding *S. mutants* and/or *C. albicans*. Bars indicate ± standard error in the linear mixed-effects model. Taxa shown are those that had FDR < 0.05 in the linear mixed-effects model. Ca—*C. albicans*, Sm—*S. mutans*, CaSm—*C. albicans* + *S. mutans*

Having detected differences in the composition and functional pathways between biofilm and planktonic phase in the simplest condition (saliva cultured under glucose/fructose supplemented condition with no added pathogens), we next sought to investigate the effects of the exogenous introduction of Sm and/or Ca to the inocula under the same condition (glucose/fructose supplemented). Regardless of the pathogen(s) added, total levels of *Streptococcus* species remained dominant, having greater than 50% relative abundance in any group (Fig. 3A). The addition of exogenous pathogens had no effect on the alpha diversity (*P* > 0.05, Fig. 3B). Beta-diversity, again measured by Bray–Curtis distances showed the exogenous pathogens to be different in biofilms but not in planktonic phases (*P* = 0.003 and 0.411 respectively, PERMANOVA test; Fig. 3C).

Seeing changes in diversity between pathogens, we compared levels of taxa that had greater than 1% mean abundance (Fig. 3D). Increases or decreases of bacterial abundance occurred mostly in the biofilm samples, except for *B. longum* which were increased both in the biofilm and planktonic phase when Sm was added (FDR = 0.006 and 0.00325, respectively). Also, *Veillonela parvula* increased in the biofilm (FDR = 0.028) only when Sm was added. The other species that increased in the biofilm was *S. salivarius* when supplemented with CaSm (FDR = 0.0437). In contrast, six taxa were decreased in the biofilm when either CaSm or Sm was added; five of six taxa were decreased in both Sm and CaSm added groups, while *Prevotella melaninogenica* decreased only in the CaSm added group and *S. pneumoniae* decreased only in the Sm added group. Interestingly, *S. parasanguinis* was the only organism that was detected at lower levels in all biofilm conditions in comparison to no external organisms added group (Saliva) (FDR = 0.0147 for Ca, FDR = 0.0059 for CaSm, and FDR = 0.0019 for Sm).

**Fig. 4:**
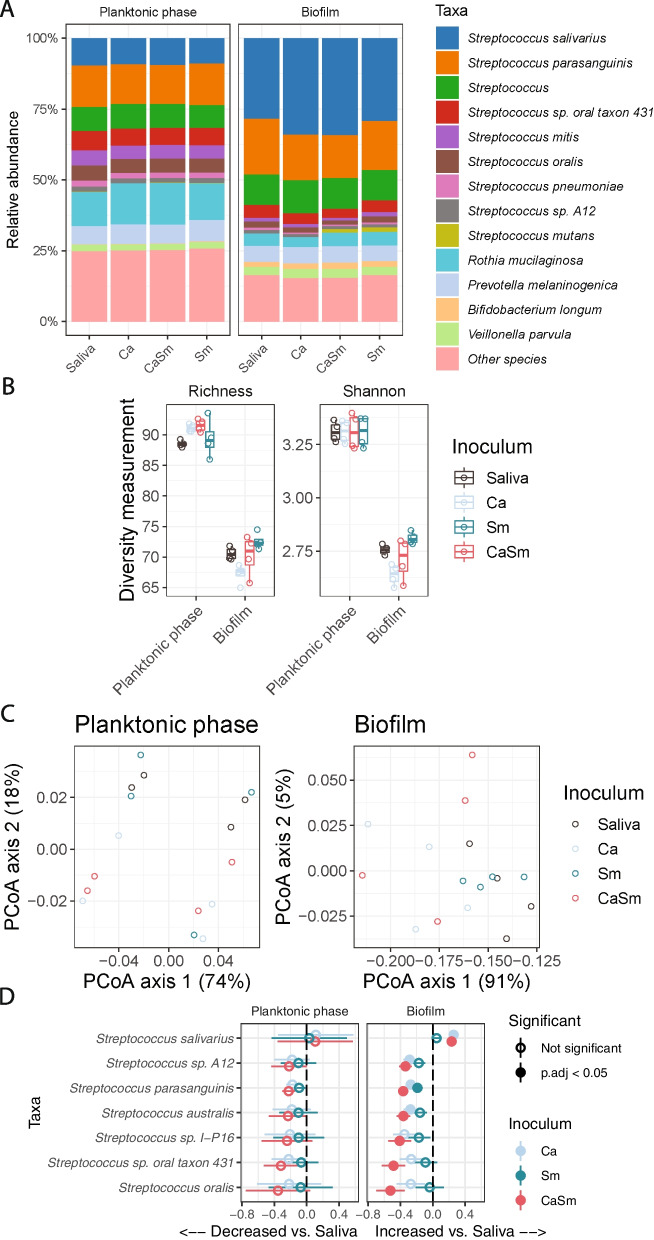
Effects of introducing opportunistic pathogens on planktonic and biofilm microbiota with sucrose. **A** Bacteria relative abundance within planktonic phases and biofilms when introducing *S. mutants* and/or *C. albicans*. **B** Alpha diversity of planktonic and biofilm samples with different pathogens. **C** Principle coordinates analysis plot of Bray–Curtis dissimilarity between samples with *S. mutants* and/or *C. albicans*. *X* and *Y* axis show the percentage of total variance captured. **D** Difference in log2-transformed abundances of bacterial species as a result of adding *S. mutants* and/or *C. albicans*. Bars indicate ± standard error in the linear mixed-effects model. Taxa shown are those that had FDR < 0.05 in the linear mixed-effects model. Ca—*C. albicans*, Sm—*S. mutans*, CaSm—*C. albicans* + *S. mutans*

Since dietary sugars are one of the most critical mediators in the pathogenesis of dental caries [2], we also performed the same analysis for sucrose-supplemented conditions. Similar to the glucose/fructose conditions, total levels of *Streptococcus* species exhibited greater than 50% relative abundance in any group, regardless of the pathogen(s) added (Fig. 4A). With sucrose as a carbon source, the indices of richness and Shannon diversity decreased significantly with the addition of Ca (*P* = 0.038 and 0.014, respectively; Fig. 4B). While alpha diversity decreased only in the Ca-supplemented biofilm samples, all exogenous pathogens added groups showed altered beta-diversity in biofilms but not in planktonic phases (*P* < 0.001 and *P* = 0.569, respectively, PERMANOVA test; Fig. 4C). Beta diversity in the biofilm was different for cultures containing sucrose or glucose/fructose (*P* < 0.001, PERMANOVA test; Figure S1A). Specifically, differences in beta diversity were detected in samples derived from cultures supplemented with Ca and/or Sm grown in the presence of either sucrose or glucose/fructose (*P* < 0.001 and *P* = 0.003, respectively, PERMANOVA test; Figs. 3C and 4C). In addition, evaluation of the impact of carbon sources on the richness between samples revealed a lower number of species-level bacterial taxa with sucrose as opposed to glucose/fructose, and likewise for Shannon diversity (*P* = 0.0063 and 0.0142, respectively; Figure S1B).

Regarding the levels of taxa that had greater than 1% mean abundance under sucrose conditions, increases or decreases of bacterial abundance occurred only in the biofilm samples, particularly those that had Ca or both Ca and Sm added (CaSm) to the inocula. The species with the largest increase in the biofilm was *S. salivarius* supplemented with Ca or CaSm under sucrose condition (FDR = 0.0063 and 0.0053, respectively, Fig. 4D), while it increased only with CaSm under glucose/fructose condition (FDR = 0.0437; Fig. 3D). Moreover, *S. oralis* had the largest decrease in biofilm samples but only in those that had both Ca and Sm added (CaSm) (FDR = 0.0252; Fig. 4D). Relative to other conditions, CaSm had the most differences in abundance (7 taxa). It is worth noting that several other species (for example, *Streptococcus* A12 and *S. australis*) decreased with CaSm but the addition of Ca or Sm alone failed to elicit a consistent effect (Fig. 4D), indicating the critical interactions between Ca and Sm in modulating taxa compositions in complex microbiota. Interestingly, only *S. parasanguinis* populations decreased under all conditions (regardless of the carbon source or addition of exogenous pathogens) when compared to the samples not containing any exogenous pathogens (Saliva) (FDR = 0.0031 for Ca, FDR = 1.03E − 4 for CaSm, and FDR = 0.0257 for Sm; Figs. 3D and 4D). Overall, the largest changes in microbial diversity and individual taxa were detected in biofilm samples supplemented with CaSm under sucrose conditions when compared to the samples containing any exogenous pathogens.

### Addition of exogenous pathogens and the carbon source modifies KEGG pathways in the biofilm

**Fig. 5:**
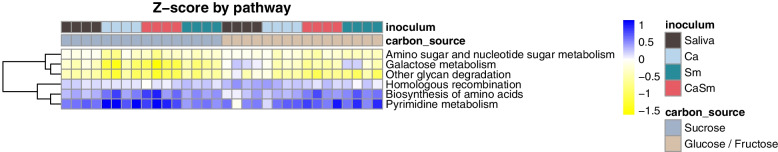
Impacts of both dietary sugars and pathogens on KEGG pathways in biofilm. *Z*-scores (scaled per pathway) of samples filtered to pathways that were differentially abundant with the addition of *C. albicans* or *S. mutans*. Pathways shown are those that had FDR < 0.05 in the linear mixed-effects model. Ca—*C. albicans*, Sm—*S. mutans*, CaSm—*C. albicans* + *S. mutans*

Finally, we assessed how KEGG pathways were modulated by the addition of exogenous pathogens and supplemented carbon sources. As our main focus was on the biofilms and the changes observed were more pronounced within the biofilms, we opted to directly compare the KEGG pathways in biofilms cultured under various conditions. Compared to Saliva condition which contains no exogenous pathogens, the addition of either CaSm or Sm resulted in comparable effects on gene expression when glucose/fructose was available as a carbon source; the biosynthesis of amino acids, amino/nucleotide sugar metabolism, and other glycan degradation pathways were influenced in the same direction by both Sm and CaSm (FDR = 0.019, 0.019, and 0.019 for Sm and FDR = 0.025, 0.029, and 0.023 for CaSm, respectively), while the homologous recombination decreased only with Sm (FDR = 0.028) and the galactose metabolism pathway decreased only with CaSm (FDR = 0.020; Fig. 5 and Table S2). In contrast, when sucrose was available as a carbon source, the increase or decrease of KEGG pathways seem more related to adding both Ca and Sm in the inoculums; the biosynthesis of amino acids pathways increased (FDR = 0.025) but the galactose metabolism and other glycan degradation pathways decreased (FDR = 0.019 and 0.025; Fig. 5 and Table S2) only with the addition of CaSm. Interestingly, the pyrimidine metabolism pathways increased both in CaSm and Ca (FDR = 0.043 for CaSm and FDR = 0.032 for Ca, respectively). In summary, while the effect of the presence of exogenous pathogens in isolation generally differed depending on the carbon source, CaSm triggered the most significant changes in KEGG pathways under sucrose conditions.

### CaSm has a greater effect on KEGG ortholog abundances with sucrose

**Fig. 6:**
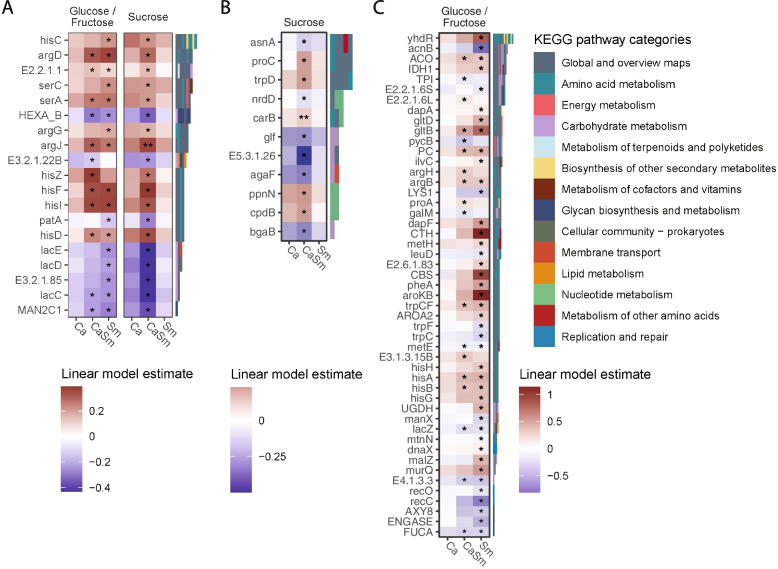
KEGG ortholog changes in prominent pathways within biofilms. **A** Heatmap of KEGG orthologs that were differentially abundant in both glucose/fructose and sucrose conditions. **B** Heatmap of KEGG orthologs that were differentially abundant only in the sucrose condition. **C** Heatmap of KEGG orthologs that were differentially abundant only in the glucose/fructose condition. Linear model estimate is based on log2-transformed relative abundances. * = FDR < 0.05. Orthologs are color-coded by their KEGG pathways categories. The total number of blocks represents the number of unique pathways each ortholog is assigned to. KEGG orthologs were selected in the pathways shown in Fig. 5. See Table S3 for a more detailed description of the orthologs

To further investigate specific KEGG orthologs among the changed pathways, we selected orthologs that belonged to pathways that were increased or decreased in either sucrose or glucose/fructose conditions. 68 orthologs changed due to the different pathogens in the glucose/fructose samples while 30 orthologs changed due to the added exogenous pathogens in the sucrose samples and there were 19 orthologs in common (Table S3). In addition to the changed pathways mentioned previously, these 19 orthologs belonged to arginine biosynthesis, lysine biosynthesis, and glycosaminoglycan degradation. The changed orthologs specific to the sucrose condition are related to the above-mentioned pathways plus alanine aspartate and glutamate metabolism, and phenylalanine, tyrosine, and tryptophan biosynthesis. However, under glucose/fructose conditions, the changed orthologs were related to a broader list of metabolism pathways including glycolysis/gluconeogenesis, fructose and mannose metabolism, ascorbate and aldarate metabolism, cysteine and methionine metabolism, and nitrogen metabolism. Among the orthologs that increased or decreased with sucrose as a carbon source, changes were mainly observed with CaSm (Fig. 6A, B). With glucose/fructose as a carbon source, all of the changes were induced with either CaSm or Sm (Fig. 6A, C). With KEGG orthologs exclusive to the glucose/fructose condition, the magnitude of change was greater with the Sm compared to CaSm; this occurred in yhdR (aspartate aminotransferase), CTH (cystathionine gamma-lyase), and aroKB (shikimate kinase) (Fig. 6C). Ca was not associated with any changes in orthologs regardless of carbon sources. In summary, significant changes were observed with CaSm under sucrose conditions that were more focused on select pathways while glucose/fructose conditions affected broader metabolic pathways in particular with Sm.

## Discussion

The pathogenesis of S-ECC is multifactorial, involving the development of cariogenic microbiota, host susceptibility, and environmental factors, especially behaviors related to the consumption of sugars [40–42] The microbial etiology of S-ECC has been framed by the longstanding dogma that *S. mutans* is a keystone species for disease development [41–43]. Recently, numerous clinical [21, 29, 44–47] and animal studies [4, 6, 7] have demonstrated an association between S-ECC and the co-infection with *S. mutans* and fungus *C. albicans* along with an increase in S-ECC severity. Those clinical studies investigated caries microbiomes using saliva or plaque samples collected from healthy or diseased subjects in a cross-sectional manner. Although these were sufficient for characterizing the bacterial composition of samples, cross-sectional studies cannot be used to determine the temporal changes in the microbiome or to identify the causal link between alterations in the composition of the oral microbiota and disease onset [22]. In addition, most mechanistic studies for such inter-kingdom interactions have been mainly studied in dual-species biofilm models using artificial media [10, 48]. While saliva-derived biofilm models exist that have been shown to maintain reproducible species and metabolic diversity [26, 49, 50], these studies also used artificial medium and did not investigate the specific roles of cross-kingdom interactions in biofilm development under conditions of different carbon sources.

The relevance of a particular model to the human condition necessitates that it simulates not only the complexity of the plaque microbiota but also the ecological conditions of the microenvironments in which the organisms are found in situ. Furthermore, to facilitate the determination of cause-and-effect relationships, a model must be reproducible, allow manipulation of microbial as well as environmental factors, and enable analyses of the large number of samples that are needed to obtain reliable data on microbiological populations [26]. Therefore, to gain a deeper ecological understanding of the role of Ca and Sm in mediating a shift in the composition of the oral microbiome, we developed a novel ex vivo biofilm model using the human salivary microbiome of a healthy donor to test whether introducing exogenous pathogens and different carbon sources can alter the microbiome composition and functionality. Given that short-read 16S rRNA gene sequencing provides limited taxonomic resolution with no functional information, in this study, we employed metagenomic shotgun sequencing. By comparing the microbiota composition in biofilms and planktonic phases supplemented with Ca and/or Sm, we demonstrated the strong influence of these organisms on the composition of the bacterial communities and their functional behavior.

Differences in microbial composition and diversity were observed in comparisons between samples derived from planktonic phases and biofilms. This outcome agreed with previous clinical findings demonstrated considerable differences in the composition of the bacterial populations isolated from saliva relative to supragingival plaque [16, 51, 52]. Notably, the addition of the organism at the initiation of cultures resulted in their successful establishment as part of the microflora in biofilms (Figure S2). Interestingly, we found lower richness and diversity in biofilm samples compared with those from planktonic phases regardless of introducing exogenous pathogens and carbon sources (Figure S3), while in clinical samples, higher richness and diversity were found in dental plaque [21, 52–54]. This could be attributed to the different conditions in the oral cavity and our model; in the current investigation, we cultured biofilms under aerobic conditions with 5% CO_2_, which could potentially reduce the number of anaerobic microorganisms. In addition to evaluating the microbial composition of our samples, we also sought to identify patterns of gene potential as a surrogate for analyzing the functional behavior of the bacterial populations isolated from biofilms and planktonic phases. As expected, the abundance of genes in KEGG pathways varied between the biofilm and planktonic microbial communities (Figure S4), which is consistent with a previous report [52].

Anaerobic fermentation is critical for the development of dental caries. Fermentation increases sugar metabolism-related pathways which serve as key markers of caries progression [55]. Among dietary sugars, sucrose has been regarded as the most cariogenic carbohydrate; it is fermentable and serves as a substrate for the production of acid and exopolysaccharides by microorganisms [2, 56]. In particular, sucrose enhances cariogenic potential in comparison with glucose and fructose by lowering the concentration of calcium, phosphorous, and fluoride [57]. Representative cariogenic bacterium, *S. mutans* can consume sucrose and starch [58] to generate acids. This requires the uptaken and transfer of sugars via phosphotransferase system (PTS); *S. mutans* encodes as many as 15 Enzyme II (EII) permeases that concomitantly phosphorylate and internalize a spectrum of mono or disaccharides [59], indicating a strong capacity to catalyze sugars. Compared with the planktonic phase, we observed elevated abundance in both starch and sucrose metabolism and PTS in biofilms (Figure S4). An increase in these pathways may be partially related to the enriched accumulation of cariogenic bacteria (such as *S. mutans*) within biofilms.

The increase in sugar metabolism-related pathways in biofilms is unsurprising but we also observed increases in other pathways that have not been described before. Within the biofilm microbiome, we observed a notable enrichment of peptidoglycan biosynthesis. Peptidoglycan is an essential bacterial cell wall component that helps to protect the integrity of cells while functioning as a scaffold that can anchor other proteins in place [60]. It is also cardinal for biofilm formation and maturation [61]. Studies have suggested various roles of peptidoglycan fragments as environmental cues in biofilm maturation, such as regulation of the germination of spores in *Bacillus subtilis* [62], productions of antimicrobial compounds by *Pseudomonas aeruginosa* [63], and the yeast-hyphal transition in *C. albicans* [64]. However, the specific function of peptidoglycan in the oral biofilm is still unclear and requires more empirical studies. Pathway for the biosynthesis of amino acids was also enriched in the communities of biofilms. Amino acids play an important role in microbial metabolism, facilitating microbial growth, biofilm formation, and biofilm dispersal [65]. Furthermore, amino acids (in the form of the enzyme) can contribute to antimicrobial resistance by modifying or inactivating drugs via enzymatic degradation; for example, amidases and acyl transferases can degrade antimicrobial drugs such as beta-lactams [65, 66]. Interestingly, we observed enriched beta-Lactam resistance in biofilms, exhibiting their enhanced severity of dysbiotic microbiome. Finally, we also observed an elevated abundance of pyrimidine metabolism pathways in biofilms, indicating that pathogenic bacterial species are actively using these pathways to support their survival and virulence in the biofilm environment [67]. Notably, the biosynthesis of amino acids and pyrimidine metabolism pathways were particularly more prominent in CaSm under sucrose conditions (Fig. 5). Previous studies demonstrated that bacteria behave differently in biofilms from the planktonic phase; mutation frequencies of bacteria [68] and horizontal gene transmissions [69] were significantly increased in biofilms. In addition, complex physiological conditions in biofilms, such as gradients of nutritions and chemicals, pH, and oxygen concentration [70, 71], might accelerate antibiotic resistance in biofilms. Along with these characteristics, the data reveal that biofilms become potentially more pathogenic and virulent when Ca and Sm are involved.

Antagonism between commensals and cariogenic bacteria is a major factor shaping the composition and virulence of dental biofilms [72]. Frequent sugar consumption can disrupt the dynamic balance of microbial homeostasis and promotes the development of virulent cariogenic biofilms [56]. For example, highly acidogenic and aciduric pathogens like Sm rapidly ferment dietary sugar and create an acidic environment that is inhibitory to the growth of beneficial commensal organisms [73–75]. Moreover, they have the capability to synthesize a variety of bacteriocins (e.g., mutacins) which grants them a competitive advantage over health-associated commensals [76]. Conversely, certain oral commensals, such as *S. parasanguinis*, *S. australis*, and the highly arginolytic clinical isolate *Streptococcus* sp. A12, can moderate acidic pH through arginine metabolism via the arginine deiminase system (ADS) [75–78]. Such alkali production can prevent the outgrowth of caries-causing pathogens and shift the chemical balance [79]. In addition to neutralization of environmental pH, other streptococcal species, such as *S. oralis*, can also secrete “chemical weapons” including hydrogen peroxide (H_2_O_2_) which can suppress the growth of *S. mutans* and help maintain a symbiotic microbiome [80]. However, it has remained unclear how the ecological stress imposed by different sugars modulates the entire oral microbial community and their functions when key pathogens (i.e., Ca and Sm) are added.

In the current study, we found that the alpha diversity of biofilms cultured using saliva without added exogenous pathogens was lower when sucrose was supplemented compared with glucose/fructose (Figure S5). When exogenous pathogens were added, not surprisingly, we found that each sugar type affected the biofilm microbial ecology differently. Under the glucose/fructose condition, most of the non-mutans streptococci were depopulated when Sm was added to the inoculum (Fig. 3D). However, in the presence of sucrose, the addition of Sm alone only reduced the abundances of *S. parasanguinis* (Fig. 4D); those health-associated commensals (i.e., *S. australis* and *Streptococcus* sp. A12) were depopulated when Ca was added to the inoculum and the reductions were more pronounced with the CaSm group. In particular, a significant reduction of *S. oralis* (a representative antagonistic strain against *S. mutans*) was observed only in biofilm samples from the CaSm group. Oppositely, the abundance of *B. longum* significantly increased with Sm in the presence of glucose/fructose but not in sucrose conditions (Figure S1C). Previous studies found a significant increase in biofilm formation and enamel demineralization when Sm was cocultured with *B. longum* and* B. animalis* [81, 82]. Species of the genus *Bifidobacterium* are natural members of the human intestinal microbiota, providing some beneficial roles in human health (e.g., increased adaptive immune response and prevention of infections, allergies, and atopic diseases) [83]. However, *Bifidobacterium* species do not seem to have such a beneficial effect in the oral cavity where they have been detected in plaque samples from ECC patients [84, 85] as well as in the saliva where its frequency was higher in children with caries [86]. Similarly, *V. parvula*, an early colonizer of dental plaque, increased under glucose/fructose conditions in this study. Although *V. parvula* cannot directly ferment glucose or most other sugars, it can utilize lactate excreted by *Streptococci* for growth [87]. Since glucose is the principal nutrient for *Streptococci *[59, 88] and lactate is the major end-product of fermentation under conditions of glucose excess [89], it is conceivable that the cross-feed between Sm and *V. parvula* is promoted when glucose is present. In terms of gene potential, we found that the ortholog changes under sucrose conditions occurred only with CaSm. In contrast, with glucose/fructose, the changes were mainly triggered by Sm; KEGG orthologs were less changed with CaSm as compared to Sm alone. It indicates that inter-microbial interactions under different carbon sources may affect the metabolic gene characteristics distinctly. Interestingly, we observed an increase of KEGG orthologs *argJ*, *argD*, and *argG* in the arginine pathways in either sugar condition and with the addition of CaSm or Sm, but never Ca alone (Fig. 6). These genes could be increased as a response to a more acidic environment in biofilms promoted by acidogenic bacteria including Sm.

Notably, our data revealed the critical roles of synergistic interaction between Ca and Sm in the complex oral microbiota, uniquely revamping the composition and the functional pathways (KEGG), particularly under the virulent condition (sucrose-rich). The largest changes in microbial diversity and individual taxa, significant changes in KEGG pathways as well as orthologs in biofilm samples supplemented with CaSm under sucrose conditions accentuate the previously suggested hypothetical roles of the cross-kingdom synergism in the pathogenesis of dental caries. All these data warrant further investigation into their interactions in complex microbial communities and the role that sugars play in modulating the microecological balance and functions in the communities.

In addition, other *C. albicans*-streptococcal interactions have been suggested to play important roles during oral opportunistic infections [48]. For example, the antagonistic relationship between Ca and *S. salivarius*, an experimental probiotic, resulted in reductions in their adhesive ability and pathogenic potential of Ca in oral candidiasis models [90–92]. However, we demonstrated that introducing Ca (either alone or with Sm) increased the relative abundance of *S. salivarius* in biofilms (Figs. 3D and 4D). This merits further investigations into the understanding of a certain antagonistic mechanism in the complex biofilm model, which may support the development of new strategies for risk assessments and disease interventions.

Although Ca was not detected in the donor’s saliva, other fungi were found (Figure S6), strengthening the notion of a mycobiome being part of the human oral microbiome eubiotics conditions [93, 94]. Compared to the planktonic phase, biofilm harbored a higher abundance of *C. albicans*, *S. cerevisiae*, and *M. globasa* (FDR = 4.9E − 4, 1.5E − 16, and 4.2E − 3 respectively, Figure S7). In particular, the increase of *C. albicans* in biofilms was notably higher than other fungi (Figure S7), indicating *C. albicans*’ strong ability to colonize and become a biofilm resident in complex oral microbiota. In addition, a recent report showed that *M. globosa* exhibited a significant association with healthy dentition, and inhibited *S. mutans* growth in vitro [95]. Thus, further work may reveal the functional role of oral fungi and their interactions with bacteriomes. Another interesting observation in the current study is that human epithelial cells were also found in the biofilms (Figure S8) consistent with recent findings in salivary polymicrobial aggregates [96]. This indicates that a more complex and diverse community beyond bacteriome and mycobiome may be involved in the biofilm formation process, expanding the scope of the biofilm study.

Given the exploratory nature of the current study, we acknowledge that there are some limitations. For instance, the use of a single individual's microbiome may have allowed us to maintain the native community’s integrity and generate consistent results. However, we recognize that microbiota compositions may differ among different saliva donors [97]. Thus, future studies are needed to capture the variability in microcosm biofilms among different saliva donors. Also, pathogens were introduced only at the initiation of cultures. While a single inoculation led to successful colonization in biofilms in this study, their consistent presence in the planktonic phase for the entire culture period might result in different outcomes. In addition, tracking temporal changes in this model via longitudinal sampling with a longer duration might more effectively explain the assembly of microbial communities as well as their compositional and functional evolution over time. Finally, we employed a static model under aerobic conditions. It is likely that a dynamic system mimicking salivary flow [98], under both aerobic and anaerobic conditions, will better represent the physiological characteristics found in the oral cavity.

## Conclusions

In summary, we developed a unique and pragmatic ex vivo biofilm model combined with metagenomic sequencing technology to analyze the constitution and function of the oral microbiome. In this proof-of-principle study, we collected saliva from a healthy donor whose saliva contained neither Ca nor Sm and introduced them individually or in combination in the presence of different sugars. The results suggest (1) saliva-derived biofilm has decreased richness and diversity but increased carbohydrate metabolism relative to the planktonic phase, (2) addition of Ca and/or Sm to native microbiome drives significant changes in its bacterial composition, (3) carbon source modifies the effect of Ca and/or Sm on microbiome diversity and taxonomic abundances, (4) addition of Ca and/or Sm affect the KEGG pathways and orthologs in biofilms distinctly depending on carbon sources, and (5) Ca-Sm combination in the presence of sucrose induce unique and specific changes in microbiota composition/diversity as well as specific effects on KEGG pathways in biofilms, exhibiting enhanced antimicrobial resistance and virulence factor associated gene expressions. The combination of Ca and Sm may hasten the development of a dysbiotic/cariogenic oral microbiome, converting biofilm to more virulent and pathogenic, which further supports the impact of cross-kingdom interaction in S-ECC. In spite of the acknowledged limitations of our approach, the current study establishes a foundation for furthering our understanding of the mechanisms that regulate microbial diversity and community functions in health and disease but more importantly, suggests a new direction in microbiome research. Future studies using a modified dynamic version of the model under different conditions will extend the current findings and contribute to the development of novel approaches to modulate microbial ecology and combat the development of oral disease.

## Supplementary Information

**Additional file 1:** **Figure S1.** Impactsof both dietary sugar and pathogens on biofilm microbiota. A) Principlecoordinates analysis plot of Bray-Curtis dissimilarity between sucrose andglucose/fructose samples. X and Y axis show the percentage of total variancecaptured. B) Alpha-diversity of samples with different pathogens and differentcarbon sources. C) Difference in log2-transformed abundances of bacterialspecies as a result of adding *S. mutants* and/or *C. albicans* insucrose or glucose/fructose conditions. Bars indicate +/- standard error in thelinear mixed-effects model. Taxa shown are those that had FDR < 0.05 in thelinear mixed-effects model. **Figure S2.** Relative abundance of (A) *S. mutans*and (B) *C. albicans* in supernatant and biofilms. **Figure S3.** Alpha-diversitydifferences between biofilms and supernatants under all conditions. (*p*-value = 4.248396e-30for shannon and 4.168968e-25 for richness). **Figure S4.** Difference inlog2-transformed abundances of KEGG pathways in biofilm compared to the supernatantunder all conditions. Bars indicate +/- standard error in the linearmixed-effects model. **Figure S5**. Alpha-diversity differences in biofilms betweensucrose and glucose/fructose conditions. (*p*-value = 0.085 for Shannon and 0.067for richness). **Figure S6.** Relative abundance of fungal species inbiofilms. RPMM; Reads per Megabase per million mapped reads. **Figure S7.** Differencein log2-transformed abundances of fungal species in biofilm vs. supernatant.Bars indicate +/- standard error in the linear mixed-effects model. Abundance wascalculated as reads per megabase genome per million mapped reads. **Figure S8.**Confocal imaging of the biofilm morphology. The bacterial cells are labeledwith SYTO 9 (green), the *C. albicans* cells are labeled withConA-tetramethylrhodamine (blue), the human cells are labeled with DAPI (grey)and the EPS matrix is labeled with Alexa fluor 647 dextran (red). Scale bar:50µm. **Table S1.** Detection of *S. mutans* and *C. albicans*in saliva from healthy donors. **Table S2.** The changes of KEGGpathways in biofilm under different carbon sources and inoculums. **Table S3.** Thechanges of individual orthologs in biofilms under different carbon sources andinoculums.

## Data Availability

All data generated or analyzed during this study are included in this published article and its supplementary information files.

## Abbreviations

aroKB: Shikimate kinase
Ca: *Candida albicans*
CFU: Colony forming unit
CLSM: Confocal laser scanning microscopy
ConA: Concanavalin A
CTH: Cystathionine gamma-lyase
DAPI: 4′,6-Diamidino-2-phenylindole
EII: Enzyme II
EPS: Extracellular polymeric substances
FDR: False discovery rate
HA: Hydroxyapatite
KEGG: Kyoto Encyclopedia of Genes and Genomes
MSB: Mitis Salivarius agar plus Bacitracin
PTS: Phosphotransferase system
S-ECC: Severe early childhood caries
sHA: Saliva-coated hydroxyapatite
Sm: *Streptococcus mutans*
UFTYE: Ultra-filtered tryptone-yeast extract
yhdR: Aspartate aminotransferase
