# Supplementary material for: Addition of cariogenic pathogens to complex oral microflora drives significant changes in biofilm compositions and functionalities
Source: Microbiome. 2023 Jun 1;11:123. doi: 10.1186/s40168-023-01561-7 (PMC10234067; doi:10.1186/s40168-023-01561-7)
Supplement: Supplementary file 2 — Additional file 1: Figure S1. Impactsof both dietary sugar and pathogens on biofilm microbiota. A) Principlecoordinates analysis plot of Bray-Curtis dissimilarity between sucrose andglucose/fructose samples. X and Y axis show the percentage of total variancecaptured. B) Alpha-diversity of samples with different pathogens and differentcarbon sources. C) Difference in log2-transformed abundances of bacterialspecies as a result of adding S. mutants and/or C. albicans insucrose or glucose/fructose conditions. Bars indicate +/- standard error in thelinear mixed-effects model. Taxa shown are those that had FDR < 0.05 in thelinear mixed-effects model. Figure S2. Relative abundance of (A) S. mutansand (B) C. albicans in supernatant and biofilms. Figure S3. Alpha-diversitydifferences between biofilms and supernatants under all conditions. (p-value = 4.248396e-30for shannon and 4.168968e-25 for richness). Figure S4. Difference inlog2-transformed abundances of KEGG pathways in biofilm compared to the supernatantunder all conditions. Bars indicate +/- standard error in the linearmixed-effects model. Figure S5. Alpha-diversity differences in biofilms betweensucrose and glucose/fructose conditions. (p-value = 0.085 for Shannon and 0.067for richness). Figure S6. Relative abundance of fungal species inbiofilms. RPMM; Reads per Megabase per million mapped reads. Figure S7. Differencein log2-transformed abundances of fungal species in biofilm vs. supernatant.Bars indicate +/- standard error in the linear mixed-effects model. Abundance wascalculated as reads per megabase genome per million mapped reads. Figure S8.Confocal imaging of the biofilm morphology. The bacterial cells are labeledwith SYTO 9 (green), the C. albicans cells are labeled withConA-tetramethylrhodamine (blue), the human cells are labeled with DAPI (grey)and the EPS matrix is labeled with Alexa fluor 647 dextran (red). Scale bar:50µm. Table S1. Detection of S. mutans and C. albicansin saliva from healthy donors. Table [file 40168_2023_1561_MOESM1_ESM.zip › Supplemental Information_revFINAL.docx]

Supplemental Information

Addition of cariogenic pathogens to complex oral microflora drives significant changes in biofilm compositions and functionality

Yuan Liu^1,†^, Scott G. Daniel^2,†^, Hye-Eun Kim^1^, Hyun Koo^3, 4^, Jonathan Korostoff^5^, Flavia Teles^4,6^, Kyle Bittinger^2,*^, Geelsu Hwang^1,4,7*^

^1^ Department of Preventive and Restorative Sciences, School of Dental Medicine, University of Pennsylvania, Philadelphia, PA 19104, USA

^2^ Department of Gastroenterology, Hepatology, and Nutrition, Children's Hospital of Philadelphia, Philadelphia, PA 19104, USA

^3^ Department of Orthodontics, School of Dental Medicine, University of Pennsylvania, Philadelphia, PA 19104, USA

^4^ Center for Innovation & Precision Dentistry, School of Dental Medicine, School of Engineering and Applied Sciences, University of Pennsylvania, Philadelphia, PA 19104, USA

^5^ Department of Periodontics, School of Dental Medicine, University of Pennsylvania, Philadelphia, PA 19104, USA

^6^ Department of Basic & Translational Sciences, School of Dental Medicine, University of Pennsylvania, Philadelphia, PA 19104, USA

^7^ Department of Chemical and Biomolecular Engineering, College of Engineering, Yonsei University, Seoul, 03722, Republic of Korea

† These authors contributed equally to this work.

**Corresponding Authors:**

**Geelsu Hwang, E-mail:** geelsuh@upenn.edu; **Kyle Bittinger, E-mail:** BITTINGERK@chop.edu


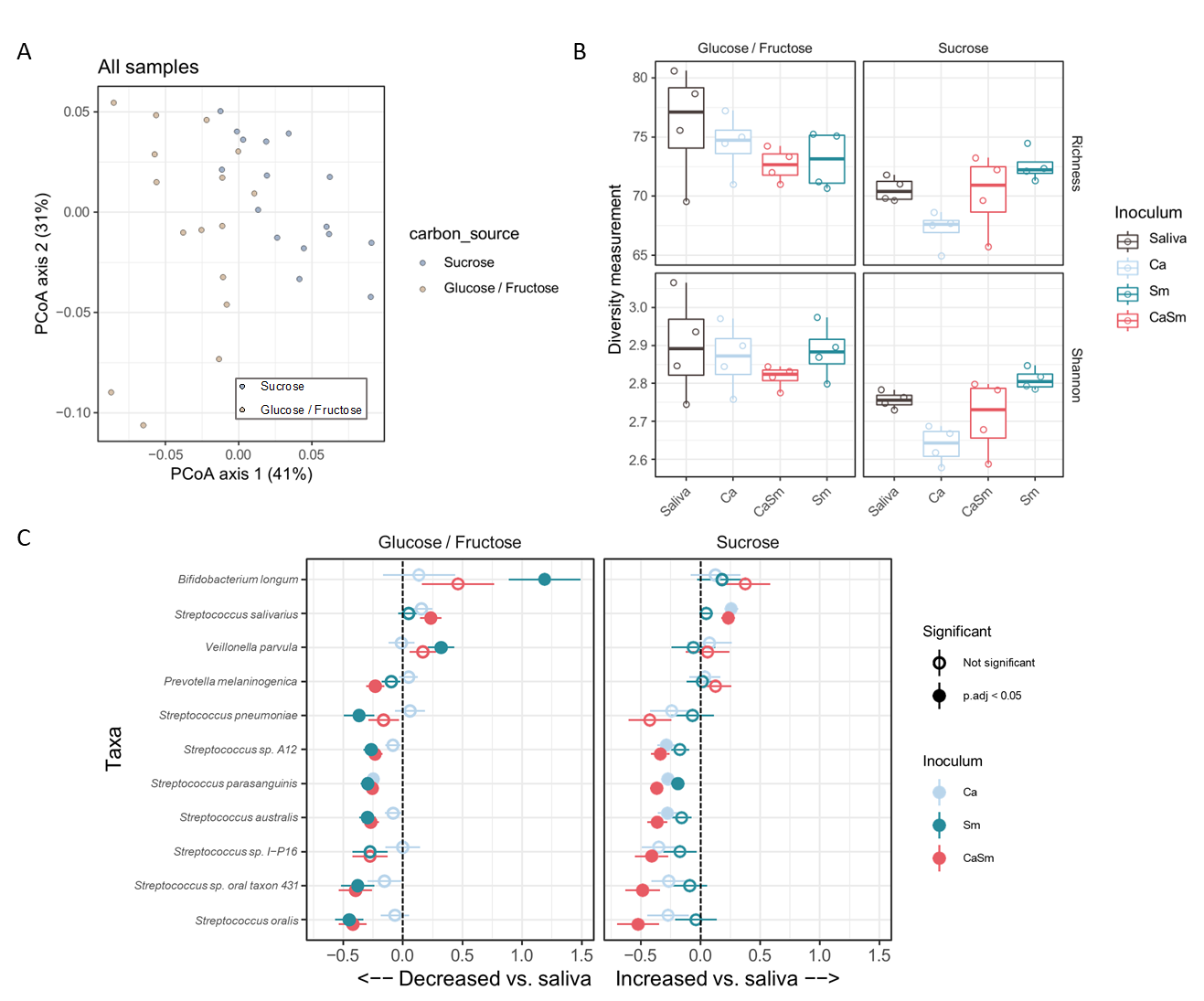


Figure S1. Impacts of both dietary sugar and pathogens on biofilm microbiota. A) Principle coordinates analysis plot of Bray-Curtis dissimilarity between sucrose and glucose/fructose samples. X and Y axis show the percentage of total variance captured. B) Alpha-diversity of samples with different pathogens and different carbon sources. C) Difference in log2-transformed abundances of bacterial species as a result of adding *S. mutants* and/or *C. albicans* in sucrose or glucose/fructose conditions. Bars indicate +/- standard error in the linear mixed-effects model. Taxa shown are those that had FDR < 0.05 in the linear mixed-effects model.


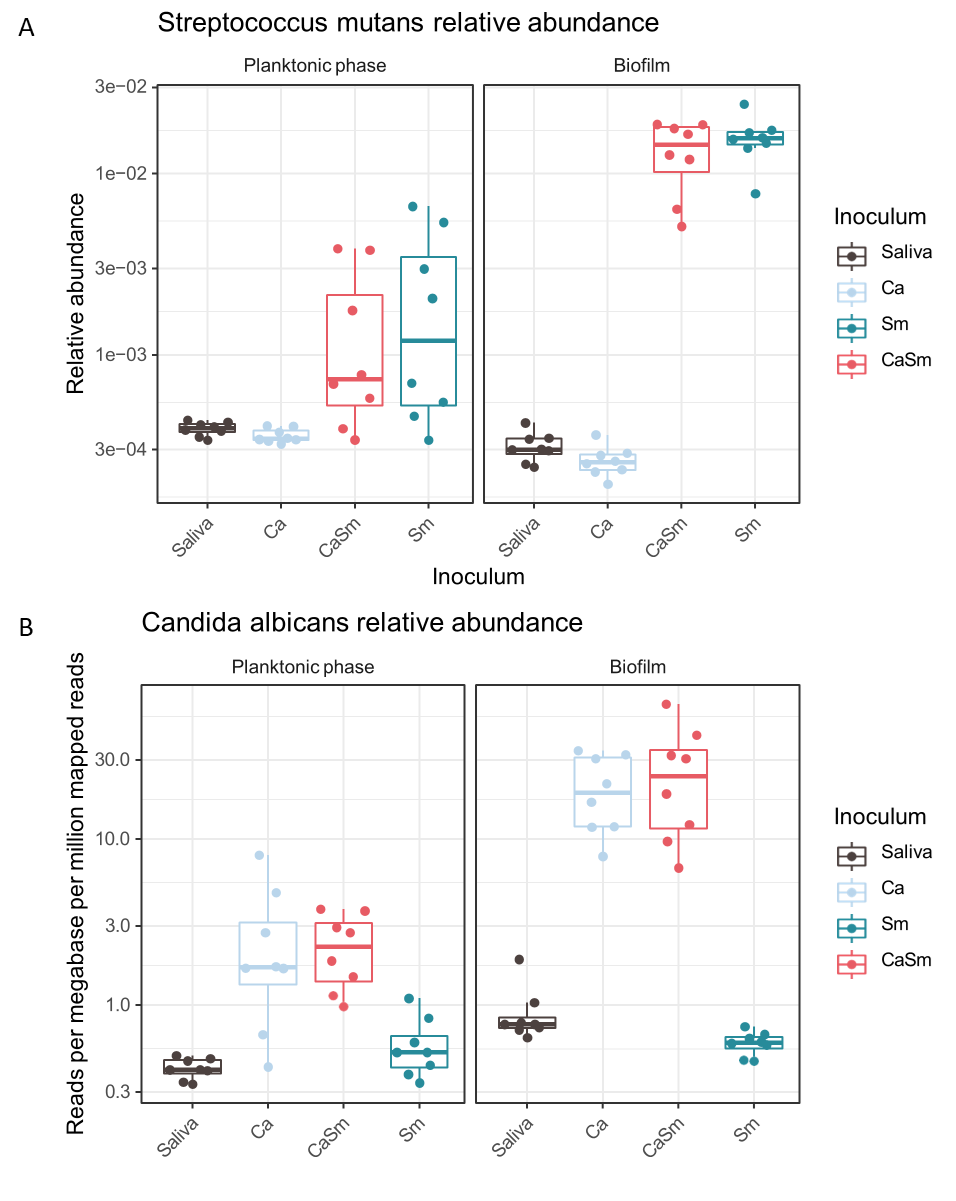


Figure S2. Relative abundance of (A) *S. mutans* and (B) *C. albicans* in supernatant and biofilms.


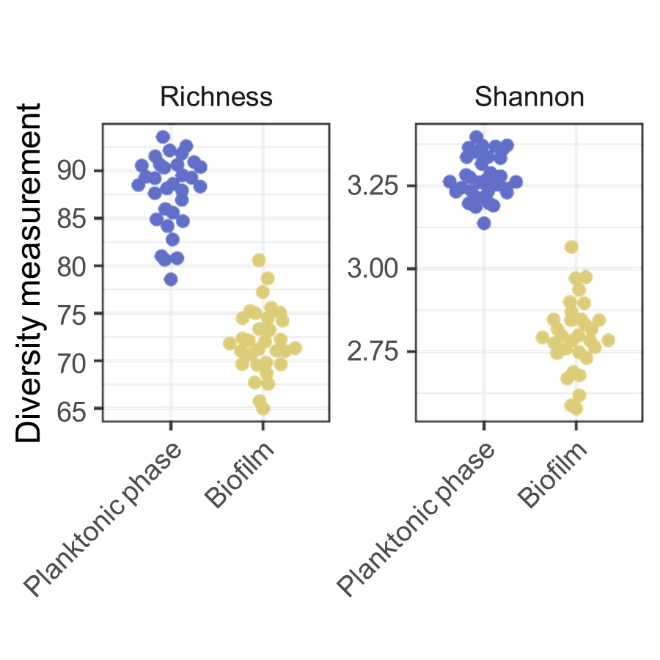


Figure S3. Alpha-diversity differences between biofilms and supernatants under all conditions. (p-value = 4.248396e-30 for shannon and 4.168968e-25 for richness)


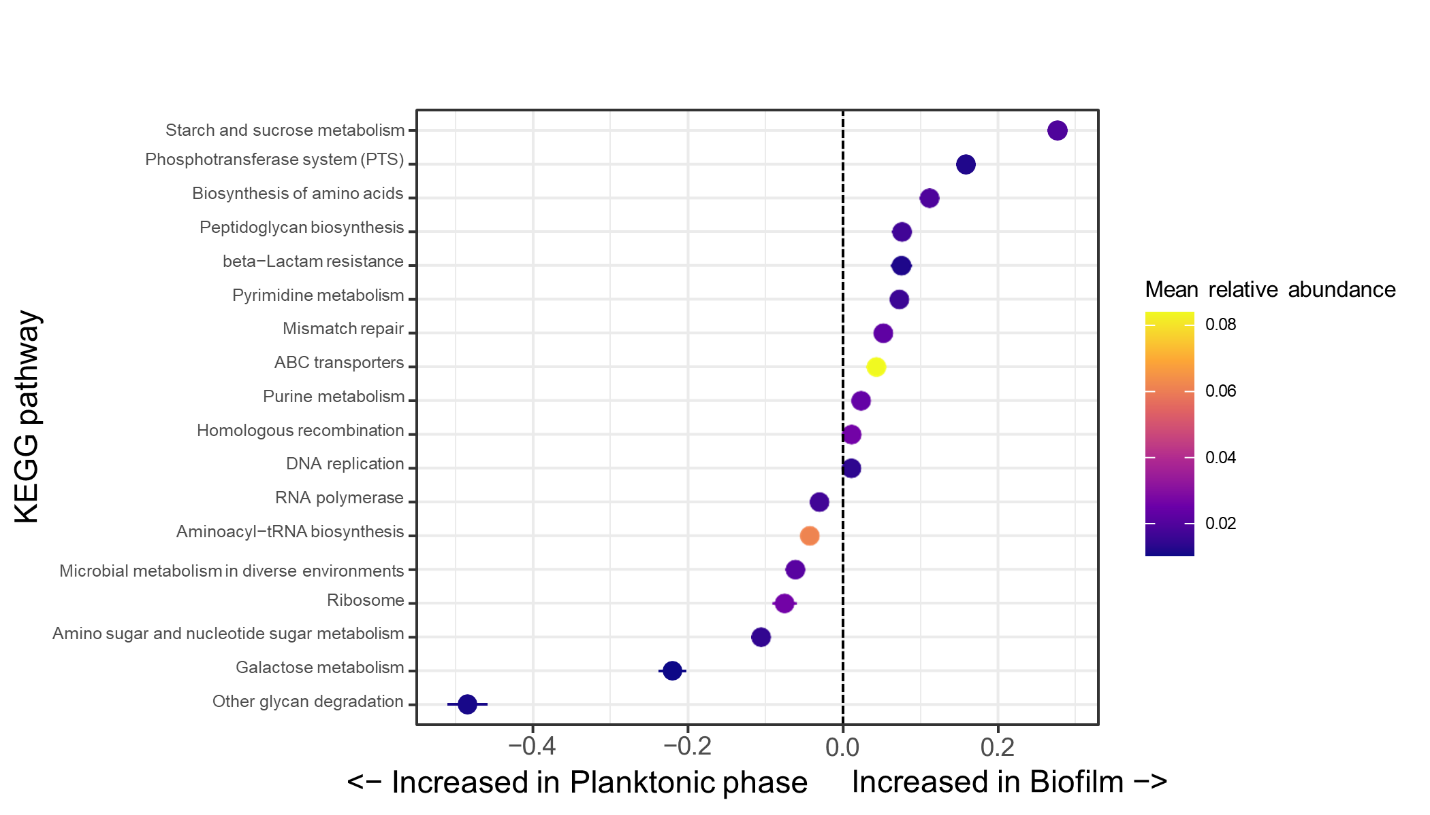


Figure S4. Difference in log2-transformed abundances of KEGG pathways in biofilm compared to the supernatant under all conditions. Bars indicate +/- standard error in the linear mixed-effects model.


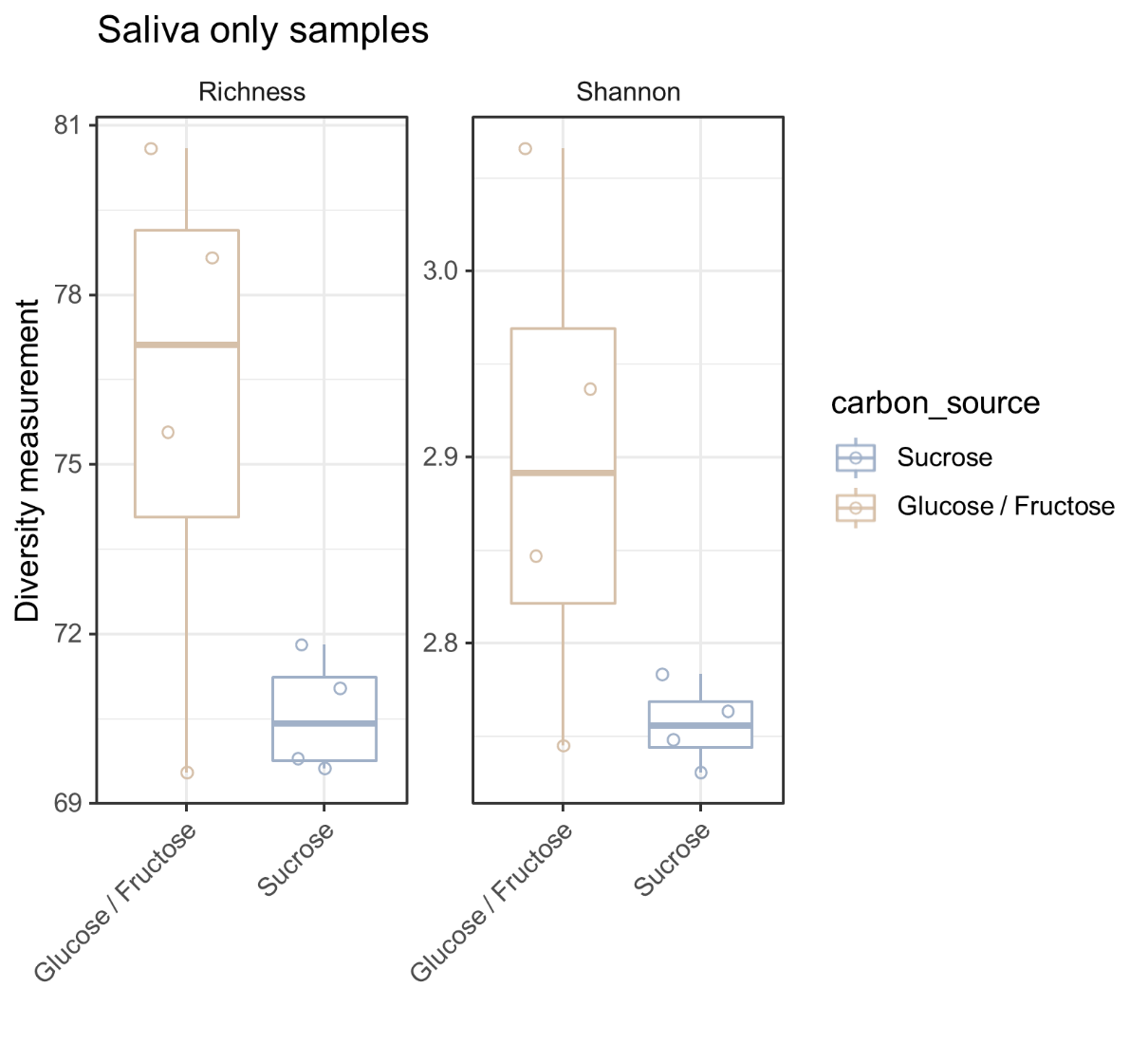


Figure S5. Alpha-diversity differences in biofilms between sucrose and glucose/fructose conditions. (p-value = 0.085 for Shannon and 0.067 for richness)


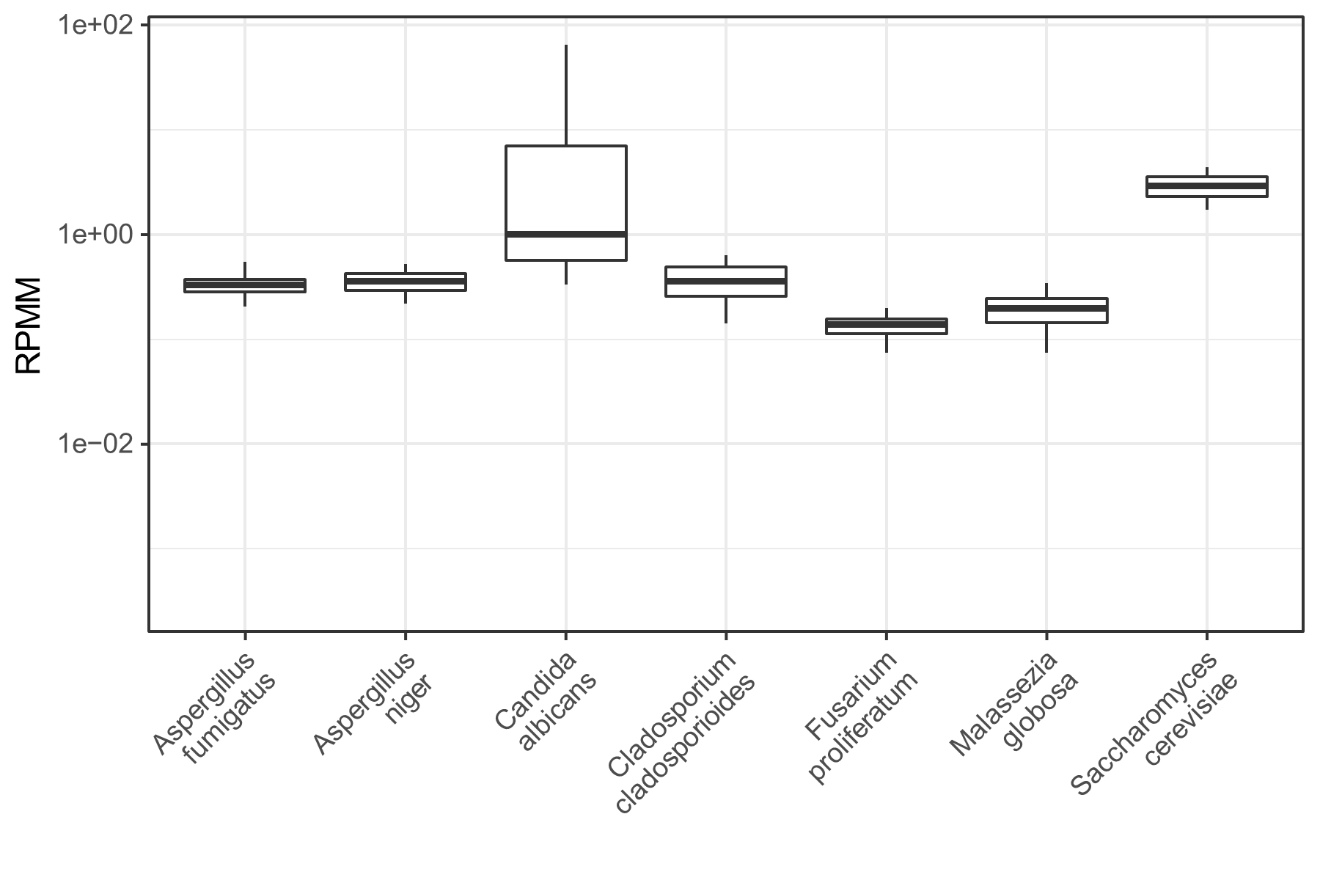


Figure S6. Relative abundance of fungal species in biofilms. RPMM; Reads per Megabase per million mapped reads


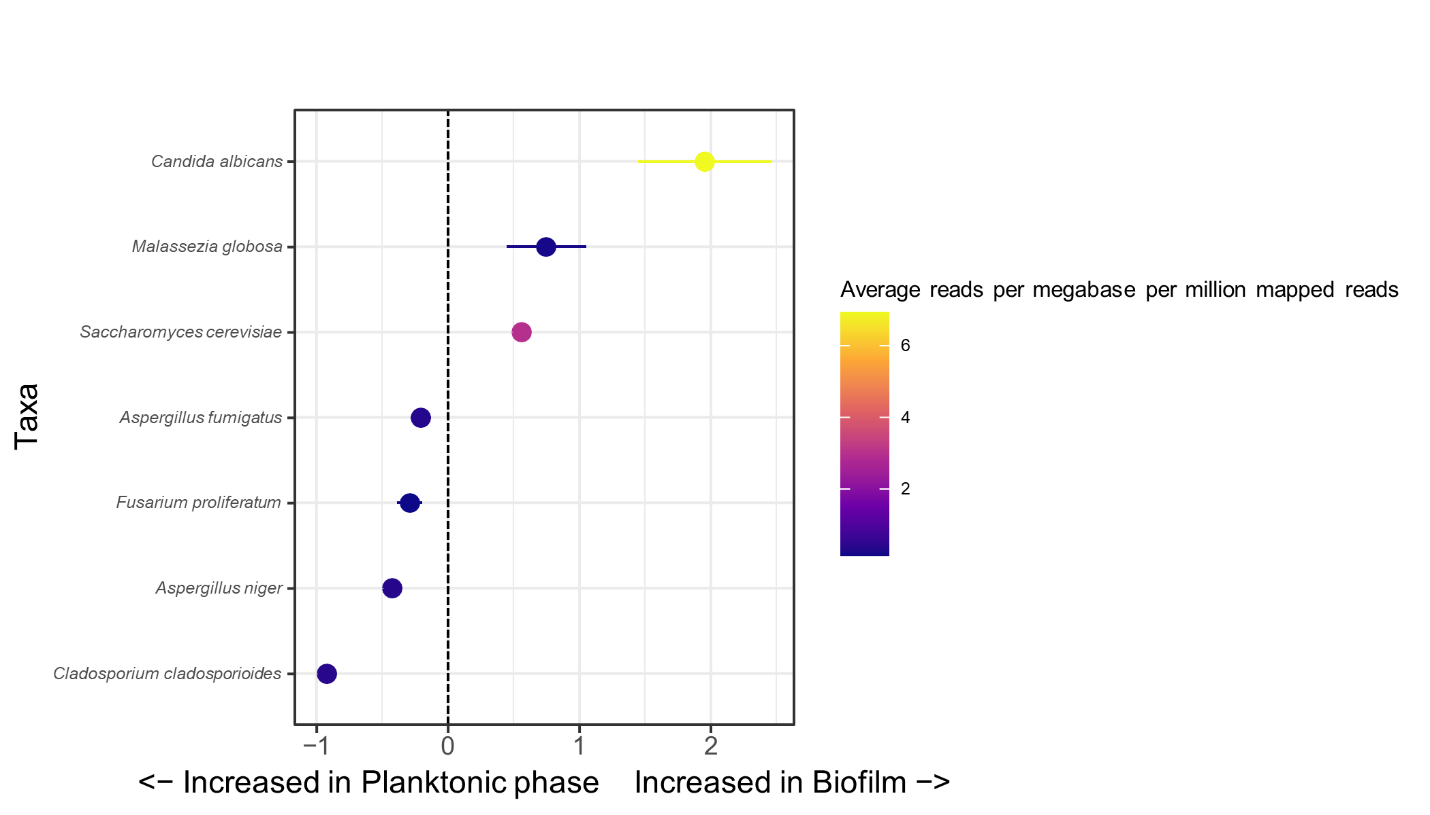


Figure S7. Difference in log2-transformed abundances of fungal species in biofilm vs. supernatant. Bars indicate +/- standard error in the linear mixed-effects model. Abundance was calculated as reads per megabase genome per million mapped reads.


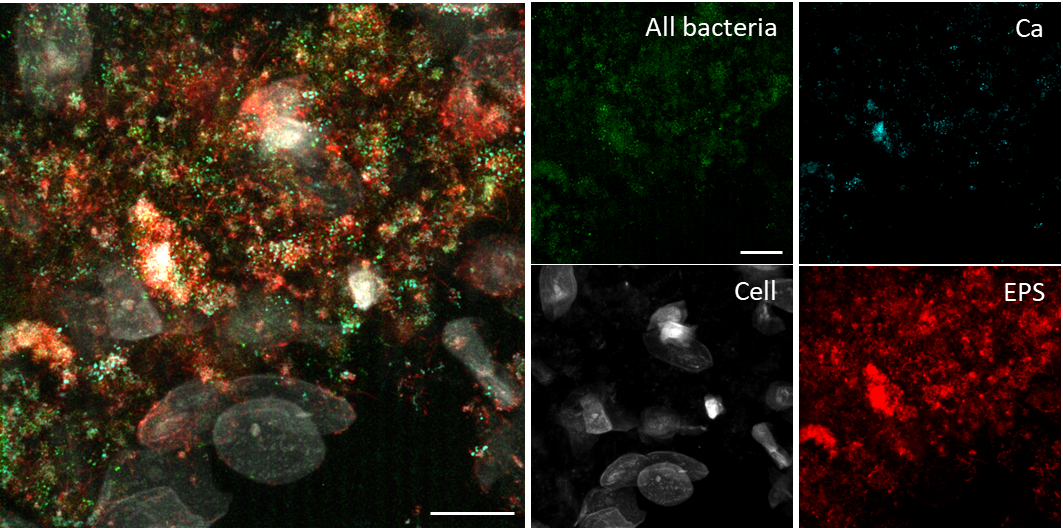


Figure S8. Confocal imaging of the biofilm morphology. The bacterial cells are labeled with SYTO 9 (green), the *C. albicans* cells are labeled with ConA-tetramethylrhodamine (blue), the human cells are labeled with DAPI (grey) and the EPS matrix is labeled with Alexa fluor 647 dextran (red). Scale bar: 50µm.

Table S1. Detection of *S. mutans* and *C. albicans* in saliva from healthy donors.

|  | *S. mutans* (10^3^ CFU/ml) | *C. albicans* (CFU/ml) |
| --- | --- | --- |
| Donor 1 | ND | ND |
| Donor 2 | 19.8±7.1 | ND |
| Donor 3 | 1.13±1.1 | ND |
| Donor 4 | 18.8±1.3 | ND |

Table S2. The changes of KEGG pathways in biofilm under different carbon sources and inoculums.

Table S3. The changes of individual orthologs in biofilms under different carbon sources and inoculums.
